# Human Foetal Neuroblasts Exhibit BK Channel‐Dependent Membrane Voltage Oscillations upon Depolarization

**DOI:** 10.1002/advs.76767

**Published:** 2026-07-24

**Authors:** Elisabetta Coppi, Federica Cherchi, Martina Venturini, Federico Tommasi, Sandro Gonzi, Chiara Capacci, Giulia Guarnieri, Pasquale Gallina, Annamaria Morelli, Anna Maria Pugliese

**Affiliations:** ^1^ Department of Neuroscience, Psychology, Division of Pharmacology and Toxicology, Drug Research and Child Health (NEUROFARBA) University of Florence Florence Italy; ^2^ Department of Neuroscience and Medical Genetics Meyer Children's Hospital IRCCS Florence Italy; ^3^ Department of Pharmacy G. D' Annunzio University of Chieti‐Pescara Chieti Italy; ^4^ Department of Physics and Astronomy University of Florence Florence Italy; ^5^ Department of Experimental and Clinical Medicine, Section of Human Anatomy and Histology University of Florence Florence Italy

**Keywords:** Ca^2+^‐activated K^+^ channels, human foetal neurblasts, intracellular Ca^2+^ rise, membrane voltage, nucleus basalis of Meynert, oscillation

## Abstract

Immature neurobalsts might present peculiar electrical activities before developing standard neuronal‐like excitability. We previously described electrophysiological properties of primary neuroblast cultures isolated from the nucleus basalis of Meynert (*hf*NBMNs) of 12‐week human foetuses, which possess the machinery for acetylcholine (ACh) synthesis, degradation and transport, functional muscarinic and nicotinic ACh receptors and tetrodotoxin‐ (TTX‐) sensitive Na^+^ and K^+^ currents. Here we report an unexpected electrical activity, encountered by serendipity in neuroblasts while seeking for standard, neuronal‐like, action potentials, consisting in high‐frequency (70 Hz on average), periodic‐like oscillations of membrane voltage evoked by cell depolarization. This activity was sensitive to intracellular thapsigargin or 1,2‐bis(2‐aminophenoxy)ethane‐*N,N,N′,N′*‐tetraacetic acid (BAPTA), as well as to extracellular tetraethylammonioum, Ba^2+^ or Iberiotoxin, thus indicating the involvement of big conductance Ca^2+^‐activated K^+^ (BK) channel family. Our data demonstrate that *hf*NBMNs present recurrent, BK‐dependent, high‐frequency voltage waves which may reflect an immature excitability pattern involved in early neuronal maturation or cholinergic network integration within foetal basal forebrain, whose lack might lead to neurodevelopmental disorders as those associated to premature birth. Further research is needed to deepen its pharmacological characterization and eventual occurrence in an integrated brain tissue.

## Graphical abstract

Primary cultures of neuroblasts isolated from the nucleus basalis of Meynert of 12‐weeks‐old human foetuses were prepared. Whole‐cell patch‐clamp recordings were performed by injecting a depolarizing stimulus current (+500 pA; 500 ms), and the membrane voltage recorded; this stimulus current evoked periodic‐like oscillations in membrane voltage (voltage waves), which were abrogated by the selective big‐conductance Ca^2+^‐dependent K^+^ channels Iberiotoxin. In the expanded trace, the mechanistic interpretation of voltage waves is reported.

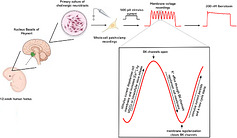

## 1 Introduction

The nucleus basalis of Meynert (NBM) represents the main source of cholinergic neurons within the basal forebrain (BF) and is a crucial player in associative learning by coordinating cortical activity and memory functions. Degeneration of NBM in humans correlates with different neurological disorders: i.e., Alzheimer's disease (AD) [1, 2, 3], Parkinson's disease (PD) [4], or long‐term cognitive impairment associated to premature birth [5]. Electrophysiological characterization of NBM neurons comes, at present, from rodent models [6, 7]. However, translation to human neurons is limited by inter‐species differences, by the conceivable complexity of the human brain and by the paucity of foetal cell source. Important experimental studies based on the use of human foetal brain tissues are trying to fill the gap between animal models and human neurons but, unfortunately, are limited in number [8, 9, 10]. Alternatively, induced pluripotent stem cells (iPSCs) [11, 12] or embryonic stem cells (ESCs) [13, 14] could be easier to access for scientific purpose but, still, results might need validation in developing “native” human neuroblasts.

We isolated neuroblasts from NBM of 12‐weeks‐old human foetuses, which correspond to the developmental period when BF nuclei are committed toward specific differentiation programs [15]. In our previous works [16, 17] we provided a first characterization of cholinergic neuroblasts (human foetal NBM neuroblasts: *hf*NBMNs) in these primary cultures and we demonstrated that they express hallmarks of cholinergic neurons, i.e., enzymes essential for acetylcholine (ACh) synthesis (choline acetyltransferase: ChAT), degradation (ACh esterase: AChE) and vesicular transport (VAChT), as well as ACh release and expression of functional muscarinic (with high levels of M2 and M3 subtypes) and nicotinic (with α5 subunit being the most abundant and β3 subunit the less) receptors [16]. We also demonstrated that *hf*NBMNs exhibit a repertoire of voltage‐dependent K^+^ currents and neuronal‐like, tetrodotoxin‐ (TTX)‐sensitive Na^+^ currents, as well as cholinergic receptor‐mediated currents [17]. Importantly, when injected in NBM‐lesioned rats, these cells promoted functional effects that could be ascribed to an improvement of the cholinergic signaling in the basal forebrain [16].

The present work is aimed to deepen the electrophysiological characterization of electrical activity in *hf*NBMNs. We observed that, when subjected to patch‐clamp recordings in the current‐clamp mode, *hf*NBMNs present unprecedented high‐frequency, periodic‐like oscillations of the membrane voltage that are sensitive to intracellular thapsigargin and 1,2‐bis(2‐aminophenoxy)ethane‐*N,N,N′,N′*‐tetraacetic acid (BAPTA) or to extracellular Iberiotoxin (IbTx), tetraetylammonium (TEA) or Ba^2+^, demonstrating the involvement of intracellular Ca^2+^ rise, voltage‐gated K^+^ (I_k_) channels and in particular big‐conductance Ca^2+^‐activated K^+^ (BK) channels.

This knowledge could be of relevance in understanding the development and functions of cholinergic neurons in the human brain, either in health or disease, particularly given that dysregulation of ion channels have been associated to CNS diseases and cognitive impairment [18].

## 2 Results

### 2.1 Human Foetal Cholinergic Neuroblasts Exhibit Recurrent Voltage Oscillations upon Membrane Depolarization

**FIGURE 1:**
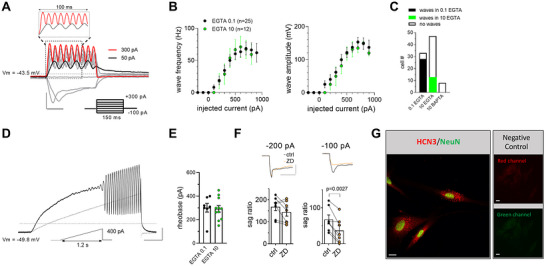
Human foetal cholinergic neuroblasts (hfNBMNs) exhibit periodic‐like voltage oscillations, i.e., voltage waves, upon depolarizing current injection. (A) Original voltage traces recorded in a representative hfNBMN where membrane voltage oscillations were observed (expanded time scale in the upper panel: 100 ms) upon depolarizing current injections (50 pA voltage steps). The dotted line indicates the 0 mV level. Scale bars: 50 ms; 100 mV. Lower panel: schematic diagram of the stimulation protocol used for current injection. (B) Averaged values (mean ± SE) of wave frequency (left panel) and amplitude (right panel) plotted vs the amplitude of injected current in cells recorded in two different experimental conditions: 10 mm EGTA‐containing (black circles, n = 25) or 0.1 mm EGTA‐containing (green circles, n = 12) pipettes. (C) Fraction of oscillating *hf*NBMNs over the total number of recorded cells in three different conditions: 0.1 mm EGTA (black column on the left), 10 mm EGTA (green central column), or 10 mm BAPTA. Empty columns in each experimental condition represent the number of cells devoid of voltage waves. (D) Original voltage traces recorded in two representative *hf*NBMNs (black trace: an oscillating cell; grey trace: a non‐oscillating cell) subjected to a depolarizing current‐ramp protocol (lower panel: from 0 to 400 pA in 1.2 s). The dotted line indicates the 0 mV level. Scale bars: 200 ms; 50 mV. (E) Pooled data of the rheobase (i.e., the minimum current to elicit voltage waves), extrapolated from the ramp protocol, in 7 *hf*NBMNs exposed to current ramps in 10 mm EGTA (black symbols) or in 11 cells in 0.1 mm EGTA (green symbols). No significant difference was found between the groups (unpaired Student's *t*‐test). (F) Pooled data of sag ratio recorded at −200 pA (left panel) or −100 pA (right panel) in control conditions (ctrl: black symbols) or after 5 min superfusion of 50 µm ZD7288 (orange symbols) in 7 *hf*NBMNs investigated (p = 0.0027, paired Student's *t*‐test). Scale bars: 50 ms; 50 mV. (G) Representative image of immunofluorescence analysis of *hf*NBMN cultures showing HCN3 (in red; NeuN in green). The adjacent negative control panel shows the red and green channels acquired after omission of the primary antibodies. Scale bar: 20.

In the present research, the electrical activity of cultured *hf*NBMNs was studied by injecting a series of increasing current steps (from −200 to +1000 pA; 300 ms duration; 2 s inter‐step interval: see lower insets in Figure 1A) in the whole‐cell patch‐clamp mode. Data reported below were obtained from 88 cells sowing an averaged C_m_ of 91.1 ± 8.4 pF, resting membrane potential (RMP) of −41.2 ± 2.1 mV, and input resistance (R_in_) of 1096.1 ± 49.6 MΩ (see Table 1).

**TABLE 1:** Passive membrane properties in *hf*NBMNs recorded in different conditions of intracellular Ca^2+^ chelation.

	C_m_ (pF)	RMP (mV)	R_in_ (MΩ)
0.1 mm EGTA oscillating (n = 28)	93.0 ± 15.0	−39.1 ± 3.7	1258.0 ± 66.2
0.1 mm EGTA not‐oscillating (n = 5)	106.5 ± 25.7	−43.4 ± 5.0	971.1 ± 135.9
p value	0.6426	0.5443	0.0397*
			
10 mm EGTA oscillating (n = 12)	74.1 ± 11.6	−39.3 ± 4.5	1014.0 ± 136.6
10 mm EGTA not‐oscillating (n = 35)	92.4 ± 20.9	−42.3 ± 4.1	1017.1 ± 102.5
p value	0.4205	0.6342	0.9855
**10** mm **EGTA‐recorded cells** p**ooled (n = 47)**	**81.7 ± 10.9**	−**43.3 ± 2.9**	**1024 ± 78.6**

10 mm BAPTA oscillating (none)	/	/	/
10 mm BAPTA not‐oscillating (n = 8)	96.0 ± 32.0	−38.8 ± 6.9	1022.2 ± 228.3

**All data pooled (n = 88)**	**91.1 ± 8.4**	−**41.2 ± 2.1**	**1096.1 ± 49.6**

C_m_: membrane capacitance; RMP: resting membrane potential; R_in_: input resistance. All p values refer to unpaired Student's *t*‐test.

In a first set of experiments, carried out by using a low‐EGTA (0.1 mm) intracellular solution, we observed an unexpected, periodic‐like, high‐frequency oscillatory activity upon membrane depolarization (Figure 1A) in the vast majority of cells (28 out of 33: 84.8%). Oscillations were defined by us “voltage waves”, and their frequency and amplitude slightly increased with increasing current injections up to a “plateau” (Figure 1B, black circles in left and right panels). Of note, even if the “plateau” phase is achieved by currents approaching the nA range (unlikely to occur in vivo), the minimum current necessary to elicit voltage waves is in the more “physiological” range of few hundreds of pA. Averaged frequency and amplitude of voltage oscillations in the 33 cells tested were 71 ± 7 Hz and 132 ± 7 mV, respectively.

In the remaining 5 cells (out of 33) where voltage waves could not be elicited, i.e., “non‐oscillating” *hf*NBMNs, we observed either: i) no electrical activity (n = 2) or ii) a single “immature” spike (n = 3) (Figure S2A), that has been already characterized in our previous work as a TTX‐insensitive spike occurring in 25.8% of *hf*NBMNs [17]. Passive membrane properties calculated in the 33 *hf*NBMNs tested are reported in Table 1. Of note, a significantly higher R_in_ was measured in oscillating (1258.0 ± 66.2 MΩ; n = 28) *vs* non‐oscillating (to 971.1 ± 135.9 MΩ; n = 5) cells (p = 0.0395, unpaired Student's *t*‐test: Table 1).

We next tested whether increased intracellular Ca^2+^ chelation, by rising intracellular EGTA from 0.1 to 10 mm, would affect voltage waves. Of note, among the 47 *hf*NBMNs recorded in 10 mm EGTA, voltage waves were detected in 12 cells (25.5%), indicating that a higher EGTA concentration in the pipette solution decreased the fraction of oscillating cells (Figure 1C: left and central columns). However, when evoked, no significant differences were observed in wave frequency nor amplitude by changing intracellular EGTA concentration (Figure 1B, green circles).

In contrast to what was observed in *hf*NBMNs recorded from 0.1 mm intracellular EGTA, when cells were recorded with 10 mm intracellular EGTA no significant differences in Rin or in other passive membrane properties were found between oscillating versus non‐oscillating cells (Table 1). Notably, when 10 mm EGTA was substituted with the more efficient Ca^2+^ chelator BAPTA (10 mm), none of the 8 cells tested presented voltage waves upon membrane depolarization (Figure 1C). Overall, the above data indicate that intracellular Ca^2+^ rise, possibly above a threshold level (not achieved in the presence of 10 mm intracellular BAPTA), is required to voltage wave initiation but, once evoked, wave frequency or amplitude are independent from intracellular Ca^2+^ chelation.

When considering the overall amount of oscillating *hf*NBMNs recorded (40 cells in total: 28 cells recorded in 0.1 mm EGTA and 12 cells in 10 mm EGTA), we extrapolated the fraction of cells expressing voltage‐dependent, TTX‐sensitive I_Na_ (Figure S2B). Of note, 11 out of the 40 oscillating *hf*NBMNs (72.5%) where devoid of I_Na_, a result which is in line with our previous work demonstrating that functional I_Na_ were recorded in the majority (i.e., 89%), but not all, *hf*NBMNs [16]. This result suggests that I_Na_ are not required to elicit voltage waves.

With the aim to establish the rheobase (i.e., the minimum injected current necessary to initiate voltage waves), a current ramp protocol (400 pA; 1.2 s: Figure 1D) was used to depolarize *hf*NBMNs. As shown in Figure 1E, a rheobase of 300 ± 40 pA (n = 7) was measured in oscillating cells recorded in 0.1 mm EGTA (Figure 1E, black symbols), a value consistent, within the experimental errors, with that obtained in 10 mm EGTA, i.e., 290 ± 30 pA (n = 12: Figure 1E, green symbols).

Hence, the average wave frequency at the rheobase value of 300 pA, as extrapolated from measurements reported in Figure 1B, is 34.5 ± 8.1 Hz in 0.1 mm EGTA (n = 25) and 28.8 ± 15.8 in 10 mm EGTA (n = 12) and these values are not statistically different (p = 0.3232, unpaired Student's *t*‐test).

At hyperpolarizing current steps, most *hf*NBMNs presented a sharp negative peak at −200 pA current injection (Figure 1F, upper left panel) and a smoother “sag‐like” voltage waveform at −100 pA (Figure 1F, upper right panel). In fact, the negative voltage peak evoked by −200 pA step current was insensitive to the I_h_ blocker ZD7288 (50 µm; Figure 1F, left panel, orange symbols and traces; n = 7) whereas, at −100 pA, a significant reduction in sag ratio was induced by ZD7288 (Figure 1F, right panel, orange symbols and traces; p = 0.0027, paired Student's *t*‐test; n = 7). Consistently with ZD7288 block, *hf*NBMNs were found to express abundant levels of the I_h_ channel subunit HCN3 (Figure 1G).

### 2.2 Voltage Waves in *hf*NBMNs are Insensitive to Voltage‐Dependent Na^+^ or Ca^2+^ Channel Blockers but are Prevented or Impaired by K^+^ Channel Block

We next investigated, by using a pharmacological approach, the current/s underpinning voltage waves in *hf*NBMNs. To this aim, cells were recorded with 0.1 mm EGTA‐containing pipettes to maximize wave occurrence. For each compound tested, wave parameters (frequency, amplitude, half width, integral) were measured and averaged.

**FIGURE 2:**
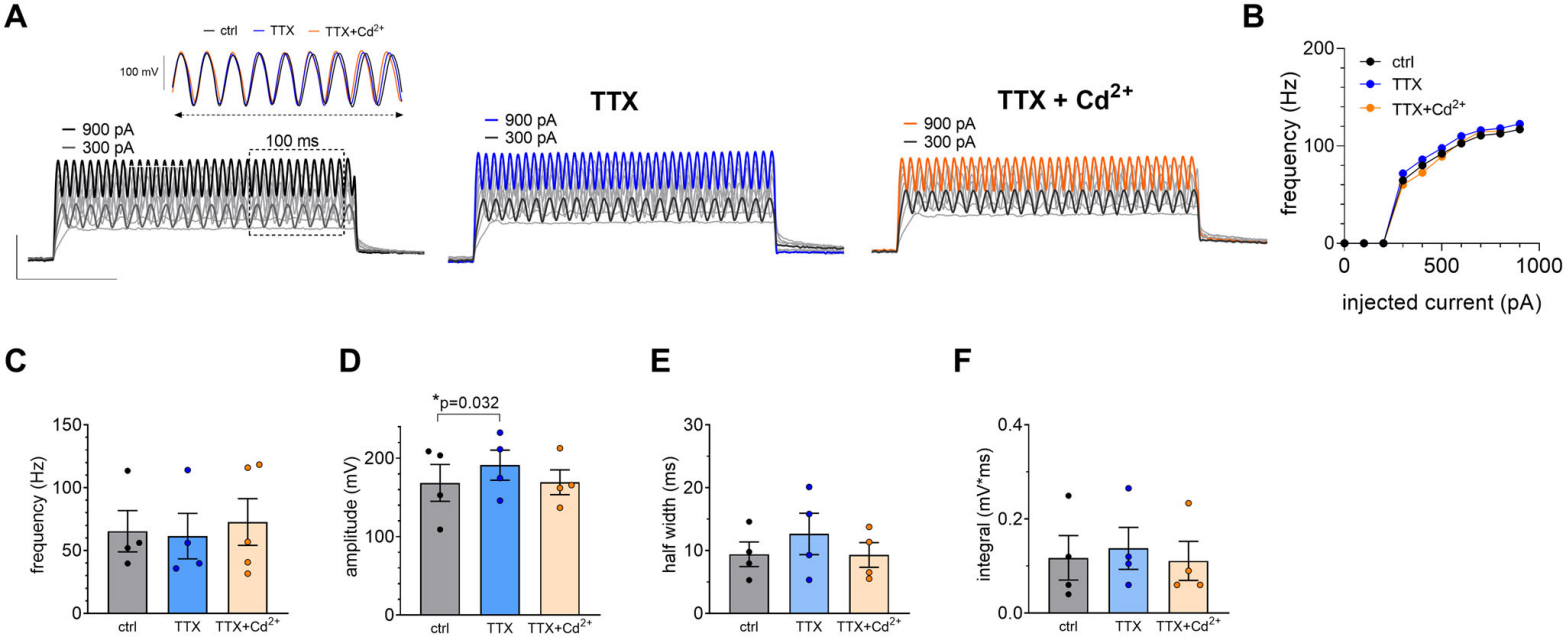
Voltage waves in *hf*NBMNs are insensitive to the voltage‐dependent Na^+^ channel blocker tetrodotoxin (TTX) or the high‐voltage activated Ca^2+^ channel blocker Cd^2+^ when applied at a concentration of 1 mm. (A) Original voltage traces recorded in a representative *hf*NBMN and evoked by increasing current values (100 pA steps) in the absence (left panel) or presence of the Na^+^ channel blocker tetrodotoxin (TTX, 1 µm) alone (central panel) or co‐applied with the Ca^2+^ channel blocker Cd^2+^ (1 mm; right panel). Scale bars: 100 ms; 100 mV. Upper panel on the left: expanded time scale (100 ms) showing the control (ctrl: black) trace; the trace recorded in 1 µm TTX (blue trace) or that recorded in 1 µm TTX + 1 mm Cd^2+^ (orange trace) at 900 pA of current injection in the same cell. (B) Wave frequency is plotted versus the injected current in the same cell. (C‐F) Pooled data (mean ± SE) of (C) wave frequency, (D) amplitude, (E) half width, and (F) integral measured in 4 cells investigated (in 0.1 mm EGTA) in ctrl (grey columns); 1 µm TTX (blue columns) or 1 µm TTX + 1 mm Cd^2+^ (orange column). *p = 0.032, paired Student's *t*‐test.

Of note, the voltage‐dependent Na^+^ channel blocker TTX (1 µm) was unable to prevent voltage oscillations (Figure 2A left and central panels), a result consistent with the above‐mentioned lack of I_Na_ in a fraction (11 out of 40) of oscillating cells. Furthermore, a subsequent application of the high‐voltage activated Ca^2+^ channel (VDCC) blocker Cd^2+^ (1 mm), still in the presence of TTX (Figure 2A: right panel) did not affect voltage waves either (Figure 2A, right panel, and 2B). Results were confirmed in 4 cells (Figure 2C–F) where neither compound modified any of the waveform parameters, except TTX that increased wave amplitude (from 169.3 ± 15.8 mV in control to 191.1 ± 19.2 mV in TTX, p = 0.0320, paired Student's t‐test, n = 4; Figure 2D).

We next tried to modulate voltage waves by targeting voltage‐gated K^+^ channels or ionotropic neurotransmitter receptors. Considering that the NBM contains cholinergic neurons that receive extensive GABAergic inputs from the striatum and amigdala [19] as primary innervation and diffuse cholinergic projections to the cortex, we tested cholinergic and GABAergic receptor ligands as well as further voltage‐dependent channel blockers.

**FIGURE 3:**
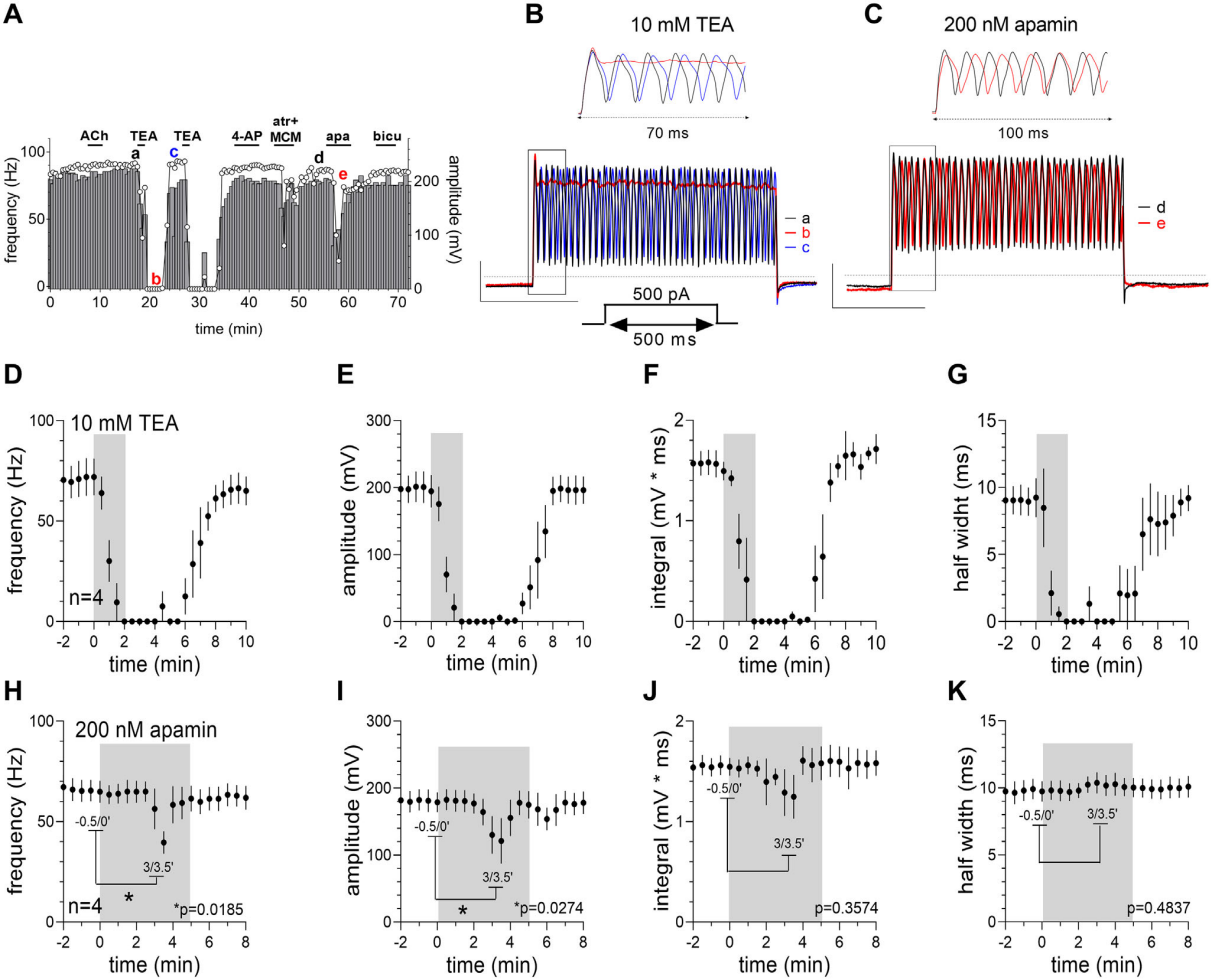
Voltage waves in *hf*NBMNs are prevented by the K^+^ channel blockers tetraethylammonium (TEA) and apamin. (A) The frequency (left y axis, in bins) and amplitude (right y axis, in dots) of voltage waves evoked by a current step of 500 pA (500 ms duration: lower inset in (B)) are expressed as a function of time in a representative *hf*NBMN exposed to different compounds: Acetylcholin (ACh; 50 µm); TEA (10 mm); 4‐amynopyridine (4‐AP; 1 mm); atropine + mecamilamine (atr + MCM; 100 nm and 10 µm, respectively); apamin (apa; 200 nm) and bicuculline (bicu; 1 µm). Letters in the graph (a, b, c, d, e) indicate specific time points chosen to compare original voltage traces. B,C. Original voltage traces recorded in the same cell at specific time points: before (a: black trace in (B)), during (b: red trace in (B)) and after (c: blue trace in (B)) the application of the nonselective K^+^ channel blocker TEA or before (d: black trace in (C)) and during (e: red trace in (C)) the application of the small conductance Ca^2+^‐activated K^+^ channel (SK) blocker apamin. Scale bars: 200 ms; 100 mV. Upper panels: expanded time scale (100 ms) of respective original traces. (D–K) Averaged time courses (mean ± SE) of wave parameters (frequency, amplitude, integral and half width) recorded in 4 *hf*NBMNs exposed to 10 mm TEA (D–G) or in 4 *hf*NBMNs exposed to 200 nm apamin (**H–K**). Numbers below data points (e.g., −0.5/0’ and 3/3.5’ in panel H) represent the time at which values were averaged for statistics. All p values refer to paired Student's *t*‐test.

Due to the paucity of cell source, we optimized experimental conditions by using a single current step protocol of +500 pA (to maximize wave occurrence) repeated once every 30 s. This protocol elicited, in those *hf*NBMNs prone to oscillate, reproducible oscillations over a relatively long‐time span (up to 30 min: Figure S3), during which different compounds could be applied and washed off consequently in a randomized sequence. One representative experiment is shown in Figure 3A, where the frequency (left‐*y*‐axis‐referring bins) and amplitude (right‐*y*‐axis‐referring open circles) of voltage waves in the absence or presence of different compounds are plotted over time. Original voltage traces recorded at specific time points are compared in Figure 3B,C. The nonselective K^+^ channel blocker TEA was the first compound we found to be able to prevent voltage waves. As shown in Figure 3A,B, oscillations completely and reversibly blocked by 10 mm TEA, and this result was confirmed in 4 cells tested (Figure 3D‐G; Table 1). The effect was fast and reversible, as repeated applications in the same cell gave reproducible results (Figure 3A). These data point to a mandatory role of voltage‐gated K^+^ currents in voltage waves.

Among the various subtype/s of K^+^ channels possibly contributing to this phenomenon, K_Ca_ were conceivable candidates, *as per* BAPTA sensitivity, of voltage waves shown above (see Figure 1C). Apamin (200 nm; 5 min application), a selective blocker of small‐conductance Ca^2+^‐activated K^+^ (SK) channels, significantly decreased wave frequency (from 66.8 ± 6.2 Hz in control to 48.3 ± 7.9 Hz in apamin, p = 0.0185, paired Student's *t*‐test, n = 4; Figure 3H) and amplitude (from 179.6 ± 8.0 mV in control to 116.9 ± 8.7 mV in apamin, p = 0.0274, paired Student's *t*‐test, n = 4; Figure 3I) and disrupted wave “periodicity” as demonstrated by the significant broadening of the frequency spectrum (Figure S5A), without significant changes in wave integral (Figure 3J) nor half width (Figure 3K). Of note, apamin‐induced effects expired before drug removal, suggesting that SK channels contribute, but are not mandatory, to voltage waves and some “adaptation” phenomena might occur to restore oscillations in case of SK channel block.

**FIGURE 4:**
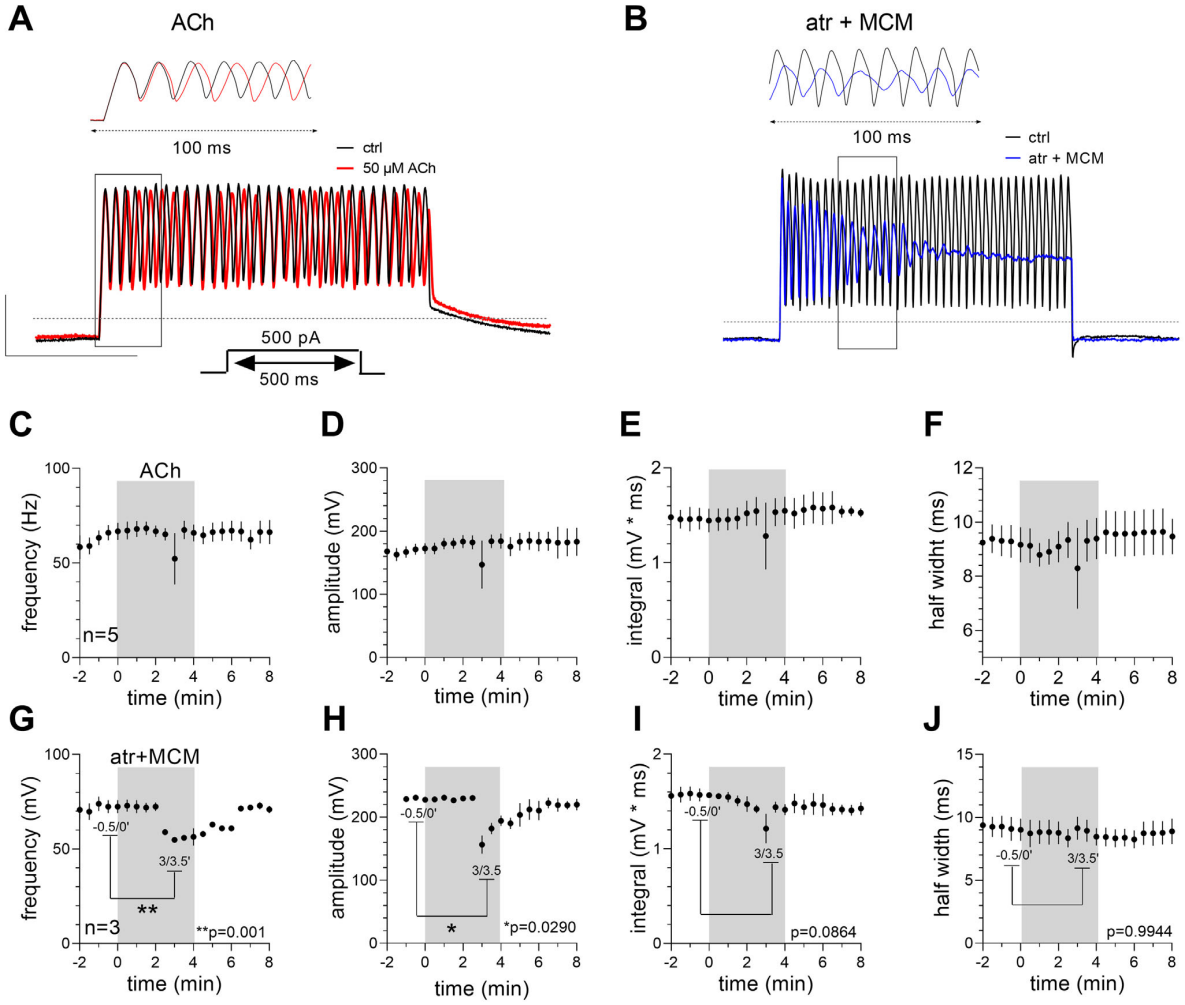
Voltage waves in *hf*NBMNs are sensitive to a combination of nicotinic plus muscarinic acetylcholine (ACh) receptor antagonists. (A,B) Original voltage traces evoked by a 500 pA current injection (lower panel) are recorded in a representative *hf*NBM before (control, ctrl: black trace in A) and during the application of 50 µm ACh (4 min application; red trace in A) or before (ctrl: black trace in B) and during the application of 100 nm atropine + 10 µm mecamilamine (Atr + MCM: 4 min application; blue trace in B). Dotted lines represent the 0 mV level. Scale bars: 200 ms; 100 mV. Upper panels: expanded time scale (100 ms) of respective original traces shown in (A,B). (C–J) Averaged time courses (mean ± SE) of wave parameters (frequency, amplitude, integral and half width) recorded in 5 *hf*NBMNs exposed to ACh (C‐F) or in 3 *hf*NBMNs exposed to Atr + MCM (G–J). Numbers below data points (e.g., −0.5/0’ and 3/3.5’ in panel G) represent the time at which values were averaged for statistics. All p values refer to paired Student's *t*‐test.

When the endogenous neurotransmitter of the NBM, ACh, was applied (50 µm; 5 min) to oscillating *hf*NBMNs, no effect on voltage wave parameters was observed (Figure 4A,C–F). This let us hypothesize that, according to our previous work demonstrating an endogenous ACh release in *hf*NBMN cultures [16], a basal, tonic, ACh receptor activation might occur in these primary cell cultures, which obscures the effect of exogenous ACh application. We next tested cholinergic receptor antagonists to corroborate our hypothesis. Of note, the combination of muscarinic plus nicotinic receptor antagonists (100 nm atropine and 10 µm mecamilamine, respectively) impaired wave maintenance during the depolarizing current step (Figure 4B,G–J), thus pointing to a contribution of cationic currents flowing through the nicotinic receptor, or voltage‐dependent currents modulated by muscarinic receptors, to voltage wave maintenance.

Conversely, anionic currents flowing through the GABA_A_ receptor are unlikely to underpin this phenomenon since the GABA_A_ receptor antagonist bicuculline (1 µm; 5 min application) did not modify oscillations (Figure 3A). However, GABA_A_ receptor antagonism depolarized *hf*NBMNs from −39 ± 2 mV in control to −25 ± 4 mV in bicuculline (Figure S5G) in the 3 cells tested, without achieving statistical significance (p = 0.1424, paired Student's *t*‐test).

**FIGURE 5:**
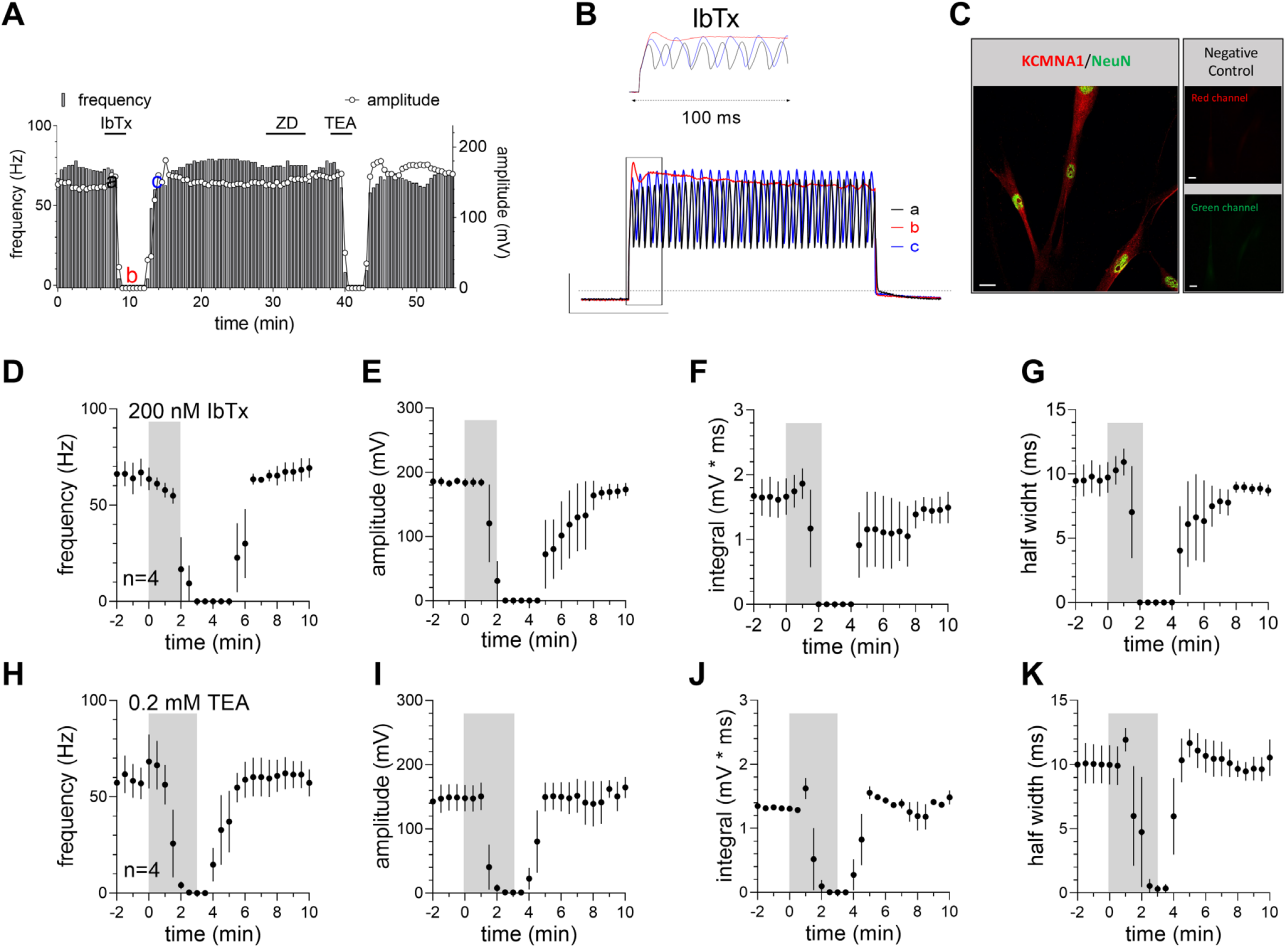
Voltage waves in *hf*NBMNs are blocked by the selective big‐conductance Ca^2+^‐activated K^+^ (BK) channel blocker iberiotoxin (IbTx). (A) The frequency (left y axis: in bins) and amplitude (right y axis: in dots) of voltage waves evoked by a current step of 500 pA (500 ms duration) are expressed as a function of time in a representative *hf*NBM exposed to different compounds: IbTx (200 nm); ZD7288 (ZD; 50 µm) or TEA (0.2 mm). Letters in the graph (a, b, c) represent specific time points where original voltage traces are compared. (B) Original voltage traces recorded in the same cell at specific time points: before (a: black trace), during (b: red trace), or after (c: blue trace) the application of 200 nm IbTx, a selective BK channel blocker. The dotted line indicates the 0 mV level. Scale bars: 200 ms; 100 mV. Upper panel: magnification of original voltage traces. (C) Representative image of immunofluorescence analysis demonstrating that *hf*NBMNs express KCNMA1 (KCa1.1: α subunit of BK channels: in red; NeuN in green). The adjacent negative control panel shows the red and green channels acquired after omission of the primary antibodies. Scale bar: 20 µm. (D–K) Averaged time courses (mean ± SE) of wave parameters (frequency, amplitude, integral and half width) measured in 4 *hf*NBMNs exposed to 200 nm IbTx (D–G) or in 4 *hf*NBMNs exposed to 0.2 mm TEA (H–K).

We then tested iberiotoxin (IbTx; 200 nm), a selective blocker of big‐conductance Ca^2+^‐activated K^+^ (BK) channels, as well as a low, sub‐millimolar (0.2 mm) concentration of TEA, considered selective for BK channel block [20]. Strikingly, both compounds abolished voltage waves (Figure 5A,B) in all cells tested (Figure 5D–K), and the effect was reversible after a few minutes of washout. Hence, these results demonstrate that, among channels underpinning voltage waves, BK are mandatory. Consistently, immunocytochemical analysis revealed the expression of BK channel α‐subunit protein in cultured *hf*NBMNs (Figure 5C).

**FIGURE 6:**
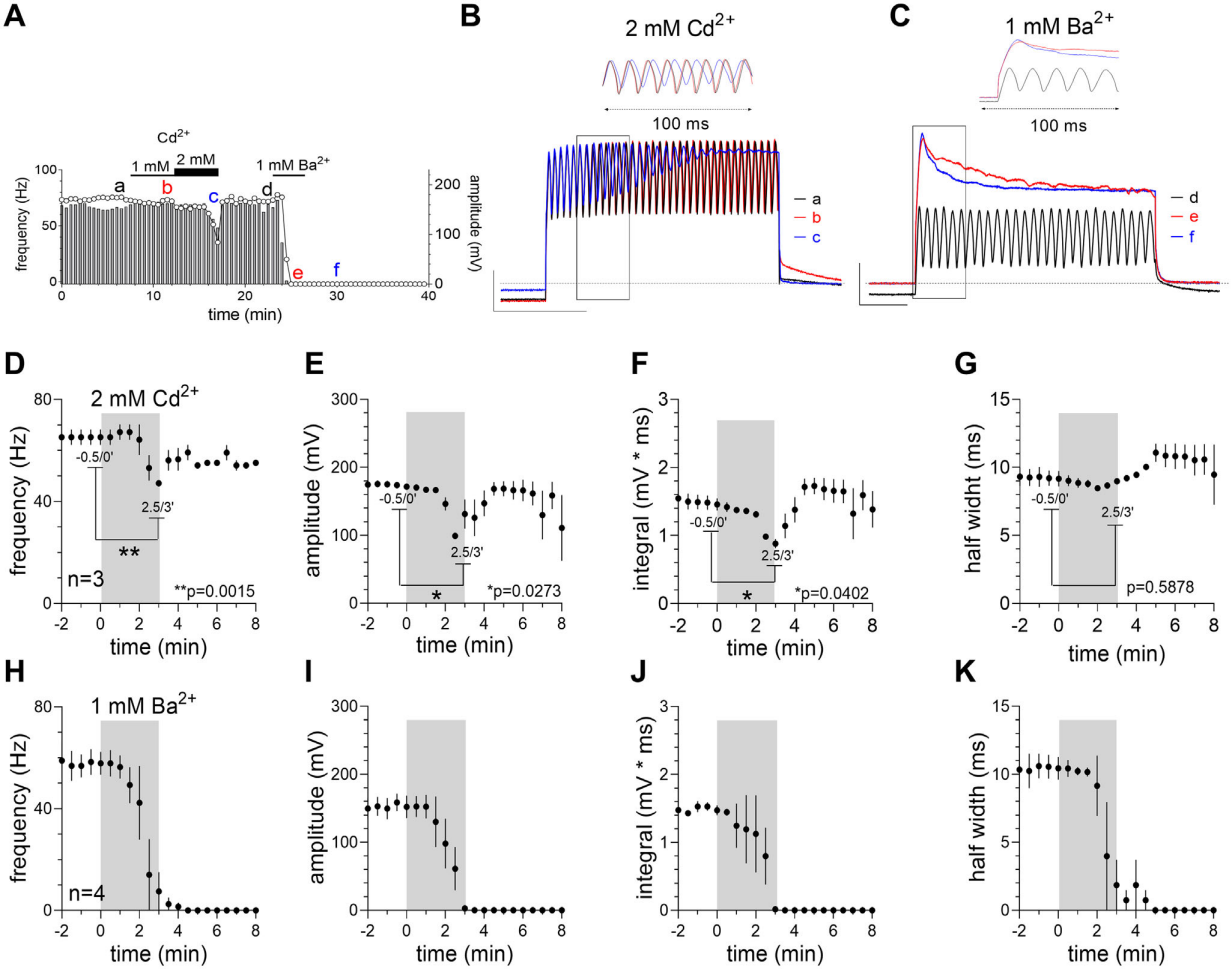
Voltage waves in *hf*NBMNs are sensitive to the high voltage‐activated Ca^2+^ channel blocker Cd^2+^ at 2 mm and are irreversibly disrupted by the K^+^ channel blocker Ba^2+^. (A) The frequency (left y axis: in bins) and amplitude (right y axis: in dots) of voltage waves are expressed as a function of time in a representative *hf*NBMN exposed to cumulative Cd^2+^ (1 and 2 mm) and Ba^2+^ (1 mm). Letters in the graph (a, b, c, d, e, f) represent specific time points where original voltage traces are compared. (B,C) Original voltage traces recorded in the same cell at specific time points: before (a: black trace in (B)), during (b: red trace in (B)) and after (c: blue trace in (B)) the application of 2 mm Cd^2+^ or before (d: black trace in C), during (e: red trace in (C)) and after (f: blue trace in (C)) the application of 1 mm Ba^2+^. Dotted lines indicate the 0 mV level. Scale bars: 200 ms; 100 mV. Upper panels: magnification of respective original traces. (D–K) Averaged time courses (mean ± SE) of wave parameters (frequency, amplitude, integral and half width) measured in 3 *hf*NBMNs exposed to 2 mm Cd^2+^ (D–G) or in 4 *hf*NBMNs exposed to 1 mm Ba^2+^ (H–K). Numbers below data points (e.g., −0.5/0’ and 2.5/3’ in panel (D)) represent the time at which values were averaged for statistics. All p values refer to paired Student's *t*‐test.

Another possible candidate would be the hyperpolarization‐activated I_h_, a conductance expressed by *hf*NBMNs as shown before (see Figure 1G) and frequently associated to recurrent electrical activity in excitable cells. Nevertheless, the I_h_, blocker ZD7288 did not affect voltage waves parameters (Figure 5A). As stated above (see Figure 2), voltage waves were insensitive to 1 mm Cd^2+^ (Figure 6A) but inhibited by 2 mm Cd^2+^ (Figure 6A,B). Results were confirmed in 4 cells tested, where a significant decrease in wave frequency (from 63 ± 3 Hz in control to 47 ± 5 Hz in 2 mm Cd^2+^; p = 0.0015, paired Student's *t*‐test, n = 3; Figure 6D), amplitude (from 174 ± 6 mV in control to 106 ± 12 mV in 2 mm Cd^2+^; p = 0.0273, paired Student's *t*‐test, n = 3; Figure 6E) and integral (from 1.5 ± 0.1 mV*ms in control to 0.9 ± 0.1 mV*ms in 2 mm Cd^2+^; p = 0.02402, paired Student's *t*‐test, n = 3; Figure 6F) were found after 3 min application of 2 mm Cd^2+^, whereas half width was unchanged (Figure 6G). Of note, 2 mm Cd^2+^ disrupted wave periodicity as it induced a significant broadening of the frequency spectrum of oscillations (Figure S4B), similarly to what observed in the presence of apamin (Figure S4A).

The K^+^ channel blocker Ba^2+^ (1 mm), irreversibly abolished voltage waves (Figure 6A,C,H–K) even after a prolonged (up to 15 min) washout (not shown), possibly because it caused massive cell depolarization (from −43 ± 6 mV in control to −12± 8 mV in 1 mm Ba^2+^; p = 0.0191; paired Student's *t*‐test, n = 3; Figure S5A), differently from TEA, apamin or IbTx, which did not affect RMP of *hf*NBMNs (Figure S5B–D, respectively). Finally, Ba^2+^ was the sole compound tested producing a rise in membrane depolarization achieved by the non‐oscillating cell membrane during wave block (Figure 6C).

### 2.3 Depletion of Intracellular Ca^2+^ Stores by Thapsigargin Transiently Impairs Voltage Waves and Confers Sensitivity to the Voltage‐Gated Ca^2+^ Channel Blocker Cd^2+^

**FIGURE 7:**
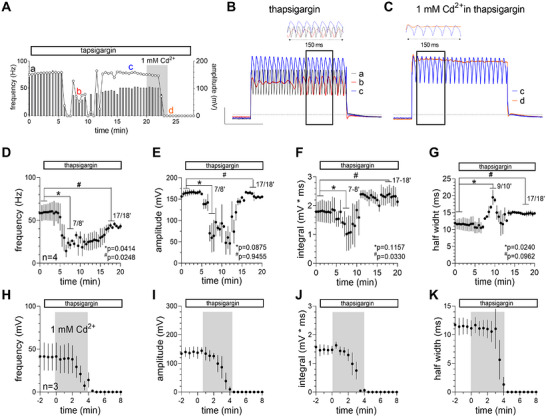
In conditions of intracellular Ca^2+^ store depletion by thapsigargin, voltage waves are significantly modified in their frequency and waveform and are irreversibly disrupted by 1 mm Cd^2+^. (A) The frequency (left y axis: in bins) and amplitude (right y axis: in dots) of voltage waves are expressed as a function of time in a representative *hf*NBMN recorded by using a thapsigargin‐ (1 µm) containing pipette and exposed to 1 mm Cd^2+^. Letters in the graph (a, b, c, d) represent specific time points where original voltage traces are compared. (B,C) Original voltage traces recorded in the same cell at specific time points: immediately after seal breakthrough (a: black trace in (B)), 8 min after seal breakthrough (b: red trace in (B)) and 17 min after seal breakthrough (c: blue traces in (B,C)), or in the presence of a subsequent application of 1 µm Cd^2+^ (d: orange trace in (C)). Dotted lines indicate the 0 mV level. Scale bars: 200 ms; 100 mV. Upper panels: magnification of respective original. (D–K) Averaged time courses (mean ± SE) of wave parameters (frequency, amplitude, integral and half width) measured in 4 *hf*NBMNs recorded with thapsigargin‐containing pipettes (D–G) or in 3 *hf*NBMNs recorded with thapsigargin‐containing pipettes and exposed to 1 µm Cd^2+^ (H–K). Numbers below data points represent the time at which values were averaged for statistics. All p values refer to One‐way ANOVA; Tukey post‐test.

Finally, in conditions of intracellular Ca^2+^ store depletion by thapsigargin (1 µm; dissolved in the pipette solution), voltage waves were transiently impaired, starting about 5 min after seal breaking through, possibly as long as the compound dialyzed the cell [21] (Figure 7A,B).

Thereafter, i.e., 8–10 min after seal breakthrough, waves recurred with broader waveform, impaired periodicity (frequency spectrum in Figure S4C) and significant changes in all measured parameters (Figure 7D–G). Under these experimental conditions, 1 mm Cd^2+^ application was sufficient to abolish oscillations (Figure 7A–C,H–K) and depolarize cells from −34 ± 7 mV to −13.8 ± 1.7 mV, even if without achieving statistical significance (p = 0.1340, paired Student's *t*‐test, n = 3; Figure S5H).

## 3 Discussion

We have previously reported a biochemical characterization and a first account of the electrophysiological properties of *hf*NBMN cultures isolated from the nucleus Basalis of Meynert of 12‐week‐old human foetuses [16, 17]. These proliferating neuroblasts express ChAT, AChE and VAChT, as well as TTX‐sensitive Na^+^ currents and immature spikes. In the present work, we provide first evidence of high‐frequency, periodic‐like, BK channel‐dependent oscillations in membrane voltage evoked by cell depolarization.

Notably, these oscillations are sensitive to IbTx, indicating that BK channels expressed by *hf*NBMN lacked the brain‐specific β4 subunit, which is typically present in BK channels from mature neurons. Thus, the immature form of BK channels seems to be instrumental to produce a susceptibility of *hf*NBMNs to maintain an oscillatory behaviour upon cell membrane depolarization, a property which is deemed to contribute to developmental processes.

The observed rhythmic, oscillatory activity of membrane voltage is, however, not to be intended as a self‐regenerating phenomenon. Indeed, the injection of a depolarizing current (the stimulus current) elicits BK channel opening that, in turn, overcomes and reverses the depolarization cause by the stimulus current. This repolarization closes BK channels by inactivation and brings the membrane potential back to the depolarized state for a new cycle, until the stimulus current is removed. In support of this mechanistic interpretation is the fact that the cell membrane time constant (τ) appears to be slower (τ ∼ 100 ms) than the oscillations (τ ∼ 10 ms peak‐to‐peak).

Hence, while oscillations display a wide range of frequencies from 5–10 Hz to about 70 Hz, according to the degree of membrane depolarization, it appears that the maximal frequency of the oscillations is likely limited by the membrane time constant, and modulated by the kinetics and number of any voltage‐dependent ion channels or ion transporters active in the cell membrane.

Of note, in those *hf*NBMNs prone to oscillate, depolarizing currents in the range of 100–200 pA are enough to elicit the oscillatory activity even if much higher values, up to 1 nA, are needed to achieve the “plateau” range of ∼70 Hz. As experiments were performed at RT (about +22°C), we should consider the fact that, in the in vivo brain at +37°C, kinetics could be faster and those depolarizations producing weak or no oscillations in vitro could have a much higher impact. Similarly, the current needed to generate the signal could be reasonable if membrane resistance is sufficiently high, as in the case of *hf*NBMNs (above 1 GΩ, see Table 1).

Interestingly, data in the literature describe an increase of total BK channel transcripts throughout the murine brain development [22] or chick cochlear development [23], where fast‐activating BK currents are known to appear at the onset of hearing [24]. Of note, as IbTx is unable to block β4‐containing BK channels [25, 26], it appears that oscillating *hf*NBMNs express β4‐devoid BK channels. The BK channel structure consists in a tetramer of pore‐forming α subunits that assemble with auxiliary β subunits up to 1:1 stoichiometry [20]. Pore channel opening requires coincident Ca^2+^ entry and membrane depolarization but, once activated, BK channels undergo rapid deactivation and inactivation, operated by auxiliary β subunits, mostly β2 and β3 [27] (for a review see: [28]). On these bases, BK channels are ideally activated during the rising phase of the AP to promote membrane repolarization and fasten the onset to subsequent AP firing, as demonstrated in rodent Purkinje cerebellar [29] or hippocampal [30] neurons where IbTx decreases the instantaneous frequency of evoked APs. The brain‐specific β4 subunit of BK channels alters channel Ca^2+^‐ and voltage‐sensitivity slowing its activation/inactivation kinetics [31] and limiting its trafficking to the cell membrane [32]. Consistently, β4 knock‐out mice exhibit a “gain‐of function” of BK channels that promotes high frequency firing (faster repolarization upon BK channel opening implies faster recovery of Na^+^ channels from inactivation), e.g., in granule cells from hippocampal slices [33], leading to the idea of disinhibited electrical activity in β4‐devoid neurons, as confirmed by the fact that β4 knock‐out mice develop spontaneous seizures [33]. By assuming a similar contribution of the β4 subunit to BK channel gating in our *hf*NBMN cultures, we might hypothesize that oscillating cells express β4‐devoid (and IbTx‐sensitive) BK channels with fast kinetics which are permissive to voltage wave initiation. Similarly to auxiliary β subunits, different isoforms of the pore‐forming α subunit might be differently assembled into the BK channel structure thereby conferring distinct voltage‐ and Ca^2+^‐dependent gating sensitivities [34]. Indeed, two different splice variants of the α subunit‐encoding *Slo* gene, namely STREX and ZERO, present regio‐specific and time‐dependent expression along embryonic mouse development [22], with predominant STREX expression in prenatal or early postnatal life [22, 34], as well as stress‐induced modulation by pituitary hormones [35]. Of note, the STREX variant represents a gain‐of‐function module that confers faster activation and slower deactivation kinetics to the BK channel, properties that would facilitate voltage wave initiation and maintenance in neuroblasts in the developing human brain as *hf*NBMNs. Overall, it appears that intrinsic biophysical properties permissive for voltage waves occurrence upon cell membrane depolarization are restricted to early neuronal development, as in our case (i.e., the first trimester of gestation of human foetal neuroblasts). This is in line with the concept of increased neuronal excitability, reflecting enhanced susceptibility to seizures, in the immature mammalian brain [36]. Conversely, the non‐oscillating *hf*NBMN counterpart might be devoid of functional BK channels on the plasma membrane or, alternatively, may express β4‐ and/or ZERO‐containing isoforms of the α subunit that, due to their gating kinetics, do not allow oscillations.

Importantly, intracellular Ca^2+^ buffering by high (10 mm) EGTA or BAPTA, decreased or abolished, respectively, voltage waves without modifying their frequency nor amplitude once elicited (in the case of sole EGTA). Hence, we conclude that depolarization‐induced intracellular Ca^2+^ rise, at least above a threshold level not easily achieved in 10 mm intracellular EGTA and never in 10 mm BAPTA, is mandatory to trigger oscillations.

Of note, K^+^ emerged as the leading ion underpinning voltage waves, as they are completely and reversibly abolished by the nonselective K^+^ channel blocker TEA (10 mm). This information, together with EGTA‐ and BAPTA‐sensitivity, gave us a clue about the involvement of K_Ca_ channels, which are key players in the conversion of intracellular Ca^2+^ oscillations into propagative electrical signals in different cell systems [37]. IbTx‐sensitivity confirmed leading BK channel involvement voltage waves, as well as TEA applied at sub‐mM concentrations, considered selective for BK channel block [38]. On the other hand, the SK channel blocker apamin decreased voltage wave frequency and amplitude, and disrupted wave periodicity, these effects being lost before apamin removal. These results demonstrate the occurrence of coping mechanisms compensating for SK channel block and indicate that SK channels, differently from BK, participate but are not mandatory to voltage wave initiation and maintenance.

Considering the intracellular Ca^2+^ source gating K_Ca_ during oscillations, the fact that the VDCC blocker Cd^2+^, applied at 1 mm concentration, did not modify oscillations in 0.1 mm EGTA and physiological‐like conditions, i.e., intact intracellular Ca^2+^ homeostasis, let us hypothesize that VDCCs are not involved in the event. However, when tested in conditions of intracellular Ca^2+^ store depletion by thapsigargin, which per se impaired and altered wave parameters (once thapsigargin dialyzes into the cell cytoplasm), 1 mm Cd^2+^ was enough to irreversibly prevented oscillations. These data demonstrate that either VDCCs or intracellular Ca^2+^ stores are potential sources to open BK channels and, indeed, they concur to produce oscillations and compensate one for each other block. However, when both Ca^2+^ sources are blocked, i.e., when 1 mm Cd^2+^ is applied in thapsigargin‐depleted cells, voltage waves are irreversibly prevented.

Beyond BK, other K^+^ channels might be involved in this phenomenon, as extracellular Ba^2+^ also prevented wave initiation. This effect, differently from IbTx or TEA, was irreversible and caused the complete loss ionic transmembrane homeostasis (see Figure S5A). Ba^2+^ is considered a relatively selective inward rectifying K^+^ channel (K_ir_) blocker when applied at submillimolar (100–200 nm) concentrations, whereas, at 1 mm as in our study, it is an nonselective K^+^ channel blocker. However, the fact that 10 mm TEA does not depolarize *hf*NBMNs, in line with the block of depolarization‐activated K^+^ channels that are closed at rest, indicates that Ba^2+^ ‐mediated effect might be due to the inhibition of TEA‐insensitive K^+^ channels opened at rest, e.g., K_ir_. It should also be noted that Ba^2+^ not only blocks voltage waves and depolarizes *hf*NBMNs to the “zero” level but also induces a higher cell depolarization upon current injection when waves are prevented (see Figure 6C). This could be due to membrane resistance increase in the presence of Ba^2+^, consistent with K_ir_ channel block, or, alternatively, to Ba^2+^ entering through VDCCs (but not able to concur to BK nor SK channel activation) and further depolarizing the cell. It is worth to note that, when BK channels were first described in the 1990's (ref. 39, 40, 41), an intracellular binding site for Ba^2+^ block was found, with an IC_50_ value at the single‐channel level as low as 200 nm [42, 43]. We cannot exclude that similar intracellular Ba^2+^ levels could be achieved when 1 mm Ba^2+^ is present in the extracellular solution. However, it still would not justify the irreversibility of Ba^2+^ effects.

In finding a functional explanation to voltage waves, it is worth to note that frequencies in the range of 20–30 Hz, as those measured in the present work at the rheobase extrapolated from ramp current stimulation (see Figure 1D,E), are reported to modulate mammalian neurodevelopment (for a review see: [43]). Similarly, intracellular Ca^2+^ spikes might promote synaptogenesis, synaptic plasticity and circuit organization [44, 45]. Hence, even if a functional explanation of this phenomenon is still lacking, we could hypothesize that, during embryogenesis, voltage waves in cholinergic precursors of the NBM might help migration throughout the developing brain, neuroblast orientation and/or organization into clusters to populate the target area and to develop the circuitry necessary to assemble into the NBM. Indeed, synchronized oscillatory network activity has long been known and extensively reported not only in rodents, i.e., spindle bursts in the neonatal rodent neocortex in vivo [46], but also in humans, i.e., delta brushes in premature human neonates [47]. Both patterns of activity disappear with maturation (for a review see: [48]), suggesting their relevance being restricted to the developmental period, as suggested by the fact that NBM neurons from non‐human adult primates have been recently reported to fire standard APs [49]. Alterations of this phenomenon, e.g. in premature birth, might lead to deficits in neural migration and circuit dysfunction, which impacts large‐scale neural dynamics and cognition processes, possibly leading to neurological impairment, i.e., schizophrenia [50], or long‐term cognitive impairment associated with premature birth [51].

From a conceptual point of view, it is from a decade that the concept of “neurons as oscillators” has been proposed in system neuroscience [52]. In fact, each time we face a regularly occurring process, it can be described as an oscillator and its complexity reduced to a mathematically tractable description in the attempt to fill the gap between single neuron electrical behaviour and signals from neuronal circuits [53].

## 4 Conclusions

In conclusion, the present work characterized the electrical activity of developing *hf*NBMNs and disclosed the presence of an oscillatory activity of membrane voltage upon cell depolarization, which is dependent on K_Ca_, mainly BK channels, and intracellular Ca^2+^ rise. Our findings are of relevance to understanding how human neuroblasts, at least those committed to NBM cholinergic neurons, behave and may provide crucial information about the development and functions of cholinergic neurons in the human brain, orchestrating circuit organization, and could help to understand how eventual dysfunctions in the cholinergic system lead to cognitive impairment or neurological symptoms in pre‐born humans.

## 5 Experimental Section

### 5.1 Cell Cultures

Two human foetuses at 12‐week gestational age were dissected 3–5 h after spontaneous or therapeutic abortion and after maternal approval. Ethic statement for the use of human foetal tissue for research purposes and study protocols used for the present research were approved by the National Ethical Committee, by the Committee for investigation in Humans and by the local University Ethical Committee (Italy; Permit Number: 678304), as already reported [54]. Fertilization ages were determined by multiple parameters, as described previously [54].The NBM was isolated as previously as reported [54]. Cell suspensions were mechanically dispersed and cultured in Coon's modified Ham's F12 medium (Euroclone, Milan, Italy) supplemented with 10% foetal bovine serum (FBS: Hyclone, Logan, UT), as described [16]. Cells were stored at −80°C and unfrozen for experimental use when needed. Culture were then growth in Coon's modified Ham's F12 medium (Euroclone, Milan, Italy; catalog no.: ECM0019L) supplemented with 10% fetal bovine serum (FBS: Hyclone, Logan, UT; catalog no.: CHA1115L) and split from plates when confluent (each split determining a culture passage, i.e., p1 represents cells split once): approximately twice a week for the first 10–12 passages (p10‐p12), then once a week/every 10 days for later passages. Experiments were performed with cells approximately from p12 to p25 (2.5‐3 months in culture), thanks to the ability of foetal cells to be maintained in culture for long periods. As demonstrated in our previous work [17], electrophysiological properties typical of immature neuroblasts were unchanged during this time span in culture.

### 5.2 Electrophysiology

Whole‐cell patch‐clamp recordings were performed as previously described [17]. The following solutions were used. Extracellular (mM): 4‐(2‐hydroxyethyl)piperazine‐1‐ethanesulfonic acid, N‐(2‐hydroxyethyl)piperazine‐N′‐(2‐ethanesulfonic acid) (HEPES) 10, D‐glucose 5, NaCl 140, KCl 3, MgCl_2_ 2, and CaCl_2_ 1 (pH 7.4 with NaOH). Pipette (mM): K‐Gluconate 130; NaCl 4.8; CaCl_2_ 1; MgCl_2_ 2; Na_2_‐ATP 5; Na_2_‐GTP 0.1; Ethylene Glycol‐bis(β‐aminoethyl ether)‐N,N,N′,N′‐tetraacetic acid (EGTA) 0.1; HEPES 10 (pH adjusted to 7.4 with KOH). The calculated free intracellular [Ca^2+^] for this pipette solution was 90 nm (Ca‐EGTA Calculator v1.3 [55]). When indicated, EGTA was raised to 10 mm and CaCl_2_ was 5 mm. The calculated liquid junction potential for this pipette solution was 15.3 mV and this value was subtracted from all current‐clamp experiments. When indicated, we raised intracellular EGTA to 10 mm or we substituted intracellular EGTA with equimolar BAPTA. Sodium currents (I_Na_) were isolated by substituting extracellular K^+^ with equimolar Cs^+^ and by using the following Cs^+^‐based pipette solution: CsCl 120; NaCl 4.8; CaCl_2_ 1; MgCl_2_ 2; Na_2_‐ATP 5; Na_2_‐GTP 0.1; EGTA 11; HEPES 10 (pH 7.4 with CsOH) in order to block K^+^ conductance. In some experiments, thapsigargin (1 µm) was added to the intracellular solution.

For whole‐cell patch clamp recordings, cells were transferred to a 1 mL recording chamber mounted on the platform of an inverted microscope (Olympus CKX41, Milan, Italy) and superfused at a flow rate of 1.5 mL/min by a three‐way perfusion valve controller (Harvard Apparatus). Borosilicate glass electrodes (Harvard Apparatus, Holliston, MA) were pulled with a Sutter Instruments puller (model P‐87) to a final tip resistance of 2–4 MΩ. Data were acquired with an Axopatch 200B amplifier (Axon Instruments, CA), low‐pass filtered at 10 kHz, stored and analyzed with pClamp 9.2 software (Axon Instruments, CA). All the experiments were carried out at room temperature (RT: 20°C–22°C). Membrane Resistance (R_m_) and membrane capacitance (C_m_) were routinely measured by fast hyperpolarizing voltage pulses (from −60 to −70 mV, 40 ms duration). When indicated, I_Na_ were evoked by a series of depolarizing voltage steps (10 mV increment from −50 to +80 mV, 40 ms duration, 2 s inter‐step interval; Vh −90 mV).

Current‐clamp recordings were performed as described [56]. Briefly, in *hf*NBMNs, we applied a series of 12 steps current injections (100 pA increment, from −200 pA to +1000 pA; 300 ms, 500 ms or 2 s duration, as indicated; 2 s inter‐episode interval) from the resting membrane potential (RMP). Sampling rate for current‐clamp recordings was 50 µs. RMP was calculated as the averaged membrane voltage measured before each current step. Sag ratio for hyperpolarizing steps was calculated as:

V_peak_—V_steady‐state_/V_peak_, where V_peak_ and V_steady‐state_ stand respectively for membrane voltage measured at the negative peak (achieved within the first 50 ms of the current step) and membrane voltage measured at the steady‐state (i.e., during the last 100 ms of the current step).

A single, 500 pA, 500 ms current step was applied, as indicated, in some *hf*NBMNs once every 30 s in the absence or presence of different compounds to obtain a time course of drug effects on voltage oscillations. Waveform analysis of voltage oscillations evoked by each current injection was performed using automatic Matlab2023a software, which identifies and counts the number of peaks in the signal, retrieving the frequency in Peaks/s (Hz), the amplitude (from the minimum to the maximum voltage reached by each peak; mV), the “half width” (i.e., the width of each peak at the half maximum voltage) and the integral (i.e., the area under the curve, AUC, of each peak, mV*ms) of each peak within an episode. Then, values obtained for each parameter measured are averaged (± Standard Error: SE) over the entire episode and plotted over time along the experimental protocol (i.e., before, during, and after the application of tested compounds). Then, the spectrum of each episode is numerically analyzed using the FFT (Fast Fourier Transform) algorithm, and the “frequency spectrum” (in Hz) is fitted with a Gaussian function to determine its broadening, which reflects the periodicity of the oscillatory events within the episode. Voltage waves were defined by us as a rhythmic, oscillatory activity in membrane voltage upon depolarizing current injection, characterized by a “frequency spectrum” between 1.5 and 4.6 Hz and, on average, 2.75 Hz (n = 40). An eventual effect of tested compounds on each wave parameter was evaluated by comparing the average of that parameter over the last 3 episodes before drug application, with the average of the same parameter over 3 consecutive episodes in the presence of the compound. See Figure S1 for the measurement of voltage wave in a single episode recorded from a typical *hf*NBMN.

Where indicated, the rheobase, defined as the minimum current value necessary to wave initiation, was quantified by injecting a depolarizing current ramp protocol (400 pA; 1.2 s) to extrapolate the minimum current value at which voltage oscillations start.

### 5.3 Immunocytochemistry

Immunofluorescence analysis was performed as previously described [57]. Briefly, the cells grown on sterile slides, were fixed in 3.7% paraformaldheyde in phosphate buffer saline (PBS) followed by permeabilization in PBS containing 0.1% Triton X‐100. Immunostaining was performed using the following primary antibodies: rabbit polyclonal anti‐HCN3 (1:80; Alomone Labs, Jerusalem, Israel), mouse monoclonal anti‐NeuN (1:100; Millipore, Temecula, CA, USA) and rabbit polycolonal anti‐KCNMA1 (KCa1.1; α subunit) (1:200; Alomone Labs) followed by Alexa Fluor 568 goat anti‐rabbit IgG (H + L) or Alexa Fluor 488 goat anti‐mouse IgG (H + L) (1:200; Molecular Probes Eugene, Oregon, USA) secondary antibodies. Antibody specificity was verified by omitting the primary antibody. Images were acquired with a confocal Leica TCS SP5 microscope equipped with a HeNe/Ar laser (Leica Microsystems, Mannheim, Germany). Observations were performed using a Leica Plan Apo 63×/1.43NA oil immersion objective.

### 5.4 Drugs

Acetylcholine (Ach), atropine (ATR), mecamilamine (MCM), BAPTA, EGTA, thapsigargin, apamin and tetraethylammonium (TEA) were purchased from SIGMA (www.sigmaaldrich.com). Iberiotoxin (IbTX) was purchased from Alomone Labs (Jerusalem, Israel). Tetrodotoxin (TTX), bicuculline and ZD7288 were purchased from TOCRIS (Bristol, United Kingdom). All drugs were dissolved in distilled water, except for IbTX, which was dissolved in PBS, stored at −20°C as 10^3^ to 10^4^ times more concentrated stock solutions, and dissolved daily in the extracellular solution to the final concentration before being applied by bath superfusion.

### 5.5 Statistics

Data are expressed as mean ± SE. Student's paired or unpaired *t*‐tests or one‐way ANOVA followed by Turkey post‐test analysis were performed, as appropriated, to determine statistical significance (p < 0.05). Data were analyzed using “GraphPad Prism 8.0.2” (San Diego, CA, USA) software or MatLab2023a software.

## Author Contributions

**Elisabetta Coppi**: conceptualization, investigation, funding acquisition, Writing – original draft, methodology, Writing – review and editing, formal analysis, project administration, data curation, supervision, resources, visualization, validation. **Anna Maria Pugliese**: validation, supervision, resources. **Annamaria Morelli**: conceptualization, validation, supervision. **Federica Cherchi**: investigation, methodology, validation, data curation. **Federico Tommasi**: conceptualization, methodology, validation, writing – review and editing, data curation, software. **Giulia Guarnieri**: methodology. Sandro Gonzi: conceptualization, validation, data curation, software. **Pasquale Gallina**: validation, resources. **Martina Venturini**: software, methodology. **Chiara Capacci**: methodology, data curation. **Chiara Capacci**: methodology, data curation.

## Funding

The present work was founded by the University of Florence, RICATEN‐2025 to EC, AMP; by Fondazione Italiana Sclerosi Multipla (FISM)—cod. 2024/R‐Single/038 to EC and financed or co‐financed with the ‘5per mille’ public founding to EC.

## Conflicts of Interest

The authors declare no conflicts of interest.

## Supporting information




**Supporting File**: advs76767‐sup‐0001‐SuppMat.docx.

## Data Availability

The data that support the findings of this study are available on request from the corresponding author. The data are not publicly available due to privacy or ethical restrictions.
