# Supplementary material for: Human Foetal Neuroblasts Exhibit BK Channel‐Dependent Membrane Voltage Oscillations upon Depolarization
Source: Adv Sci (Weinh). 2026 Jul 24:e76767. Online ahead of print. doi: 10.1002/advs.76767 (PMC13397815; doi:10.1002/advs.76767)
Supplement: Supplementary file 1 — Supporting File: advs76767‐sup‐0001‐SuppMat.docx. [file ADVS-9999-e76767-s001.docx]

**SUPPLEMENTARY MATEIAL**

**Human foetal neuroblasts exhibit BK channel-dependent membrane voltage oscillations upon depolarization**

Elisabetta Coppi^1*†^, Federica Cherchi^1,2†^, Martina Venturini^1,3^, Federico Tommasi^4^, Sandro Gonzi^4^, Chiara Capacci^1^, Giulia Guarnieri^5^, Pasquale Gallina^1^, Annamaria Morelli^5^ and Anna Maria Pugliese^1^

^1^Department of Neuroscience, Psychology, Division of Pharmacology and Toxicology, Drug Research and Child Health (NEUROFARBA), University of Florence, Florence, Italy; ^2^Department of Neuroscience and Medical Genetics, Meyer Children’s Hospital IRCCS, Florence, Italy; ^3^Department of Pharmacy, G. D' Annunzio University of Chieti-Pescara, Chieti, Italy; ^4^Department of Physics and Astronomy, University of Florence, ^5^Department of Experimental and Clinical Medicine, Section of Human Anatomy and Histology, University of Florence, Florence, Italy.

**Corresponding author:* Elisabetta Coppi, Department of NEUROFARBA, Section of Pharmacology and Toxicology, University of Florence, Italy.

elisabetta.coppi@unifi.it

^†^These authors contributed equally to this work

**Figure S1**


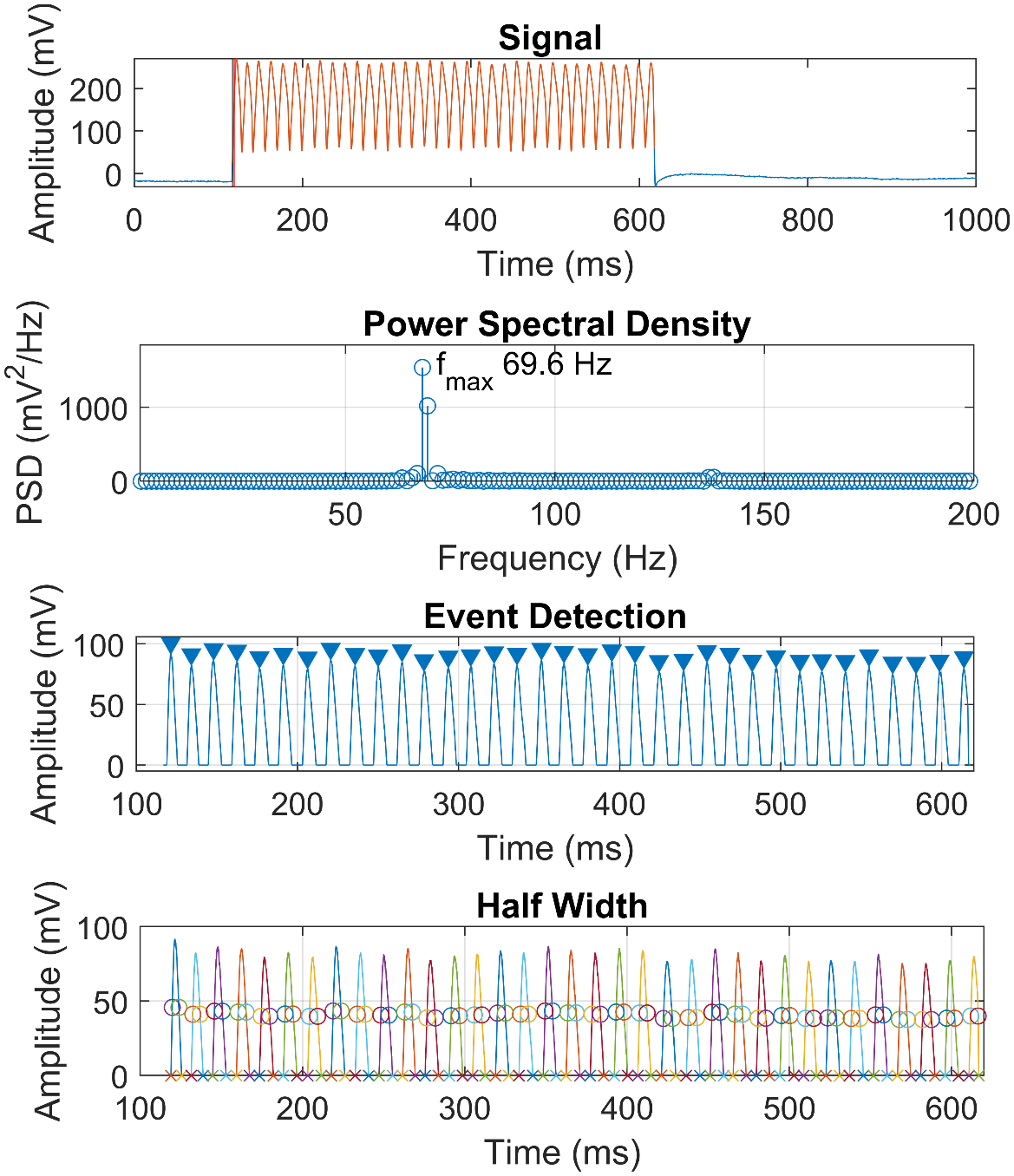


**A**

**B**

**C**

**D**

Membrane voltage (mV)

**Figure S1. Quantification of voltage waves parameters. A.** Example of analysis procedure adopted to quantify wave parameters in a representative episode evoked by a 500 pA depolarizing voltage step in a typical human foetal Nucleus Basalis of Meynert neuroblast (*hf*NBMN). The episode was analyzed using the software MatLab2023a through an algorithm, specifically generated for the present work by one of the authors (FT). **A.** Membrane voltage changes (in mV) over time (in ms) were recorded by the amplifier in the Current-clamp mode in a typical oscillating *hf*NBMN. **B.** Power Spectrum Density (PSD) analysis of the recorded signal was performed to quantify the frequency corresponding to the PSD peak (shown in the figure) and the spectral width, obtained by a Gaussian fit around the central frequency (not shown in the figure). **C,D.** Triangles **(C)** mark each detected event occurring during the episode. The algorithm automatically measures the half width of each event and its amplitude **(D)**, as well as the area under each peak (integral: not shown in the figure). Such quantities (i.e. the instantaneous frequency, the amplitude, the half width and the integral), measured for each event detected within the episode, are averaged to obtain respective values assigned to that episode, and plotted over time (one episode every 30 s) to obtain the time-course analysis of waveform parameters variation induced by the application of tested compounds (see Fig. 3). Colours represent subsequent events.

**Figure S2**

**A**

**B**

**Figure S2. Typical immature neuroblast showing a tetrodotoxin- (TTX) resistant single spike upon depolarizing current injection, despite the functional expression of TTX-sensitive Na^+^ currents. A.** Original voltage traces recorded in a human foetal Nucleus Basalis of Meynert neuroblast (*hf*NBMN) where a depolarizing current step (lower inset: +200 pA; 400 ms) elicited a single, immature spike (ctrl: black trace) insensitive to the application of TTX (1 µM; 2 min; blue trace). Scale bars: 100 mV; 200 ms. **B.** Original current traces of voltage-dependent Na^+^ currents (I_Na_; *left panel*), and respective I-V plot: *right panel*) recorded in the same cell upon the application of a depolarizing voltage step protocol (lower inset: from -50 to +80 mV; 10 mV steps of 80 ms duration) before (black circles) or after (blue circles) 2 min application of 1 µM TTX. Scale bars: 2 nA; 20 ms.

**Figure S3**

70 ms

**Figure S3. Reproducible voltage waves can be consistently evoked over a relatively long timespan (i.e. 30 min) by a saturating stimulation protocol. A.** The frequency (left *y* axis-referred bins; in Hz) and amplitude (right *y* axis-referred dots; in mV) of voltage waves evoked by a current step of 500 pA (500 ms duration: *lower inset* in **B**) are expressed as a function of time in a representative *hf*NBMN over a 30 min recording, a time span which allows reproducible signals susceptible to eventual pharmacological manipulation. **B.** Original voltage traces recorded in the same cell after 3 min (a: black trace) or 30 min (b: orange trace) recording. The dotted line represents the 0 mV level. The resting membrane potential in this cell was -39.7 mV. Scale bars: 100 mV; 200 ms.

**Figure S4**

**A**

**B**

**C**

**Figure S4. The frequency spectrum of voltage waves, as a measure of periodicity, is significantly broadened by pharmacological manipulations of Ca^2+^-dependent signals.**

**A-C.** The frequency spectrum (obtained by MatLab2023a software analysis: see Methods and Fig. S1) of voltage waves evoked by a current step of 500 pA (500 ms duration) are averaged (mean ± SE) and expressed as a function of time in cells subjected to different pharmacological manipulations: SK channel block by apamin (200 nM: **A**); high voltage-activated Ca^2+^ channel block by Cd^2+^ (2 mM; B) or intracellular Ca^2+^ store depletion by thapsigargin (1 µM: **C**). All *p* values refer to the Student’s paired t-test.

**Figure S5**

**A**

**B**

**C**

**D**

**E**

**F**

**G**

**H**

**Figure S5. Resting membrane potential (RMP) alterations in oscillating *hf*NBMNs exposed to different compounds. A-H.** Time courses of averaged (mean ± SE) RMP measured in *hf*NBMNs before, during or after the application of different compounds: the K^+^ channel blockers Ba^2+^ (1 mM; **A**) and tetraetylammonuim (TEA, 10 mM; **B**), the selective small-conductance Ca^2+^-activated K^+^ (SK) channel blocker apamin (200 nM: **C**); the selective big-conductance Ca^2+^-activated K^+^ (BK) channel blocker iberiotoxin (IbTx, 200 nM: **D**); a combination of the muscarinic receptor antagonist atropine (Atr; 100 nM) and the nicotinic receptor antagonist mecamilamine (MCM; 10 µM) (**E**); acetylcholine (ACh, 50 µM: **F**); the selective GABA_A_ receptor antagonist bicuculline (1 µM; **G**) or the intracellular Ca^2+^ store depletor thapsigargin (1 µM in the pipette solution: **H**). All *p* values refer to the Student’s paired t-test.
